# THE COMBINED IMPACT OF MIDLIFE AND LATE-LIFE VASCULAR RISK FACTORS ON 33-YEAR INCIDENT DEMENTIA: THE ARIC STUDY

**DOI:** 10.1093/geroni/igae098.2133

**Published:** 2024-12-31

**Authors:** Jason Smith, James Pike, Pamela Lutsey, Richey Sharrett, Alden Gross, Jennifer Deal

**Affiliations:** Johns Hopkins University, Baltimore, Maryland, United States; New York University, New York, New York, United States; University of Minnesota, Minneapolis, Minnesota, United States; Johns Hopkins University, Baltimore, Maryland, United States; Johns Hopkins Bloomberg School of Public Health, Baltimore, Maryland, United States; Johns Hopkins University, Baltimore, Maryland, United States

## Abstract

Midlife vascular risk factors are associated with an increased risk of dementia. However, the overall contribution of modifiable vascular risk factors in midlife and late-life to dementia remains unclear. In this study, we quantified population attributable fractions, which account for risk factor prevalence and strength of relative risks, of incident dementia from vascular risk factors measured in midlife and early late-life. We used 33 years of prospective cohort data in the Atherosclerosis Risk in Communities (ARIC) Study. Incident dementia was ascertained from baseline (1987-1989) through December 31, 2020. We quantified population attributable fractions of incident dementia by age 80 from the combination of hypertension, diabetes, and self-reported current cigarette smoking (at least one risk factor versus none) measured at different ages (45-54 [n=7,731], 55-64 [n=12,273]; 65-74 [n=6,660]). Overall, the prevalence of having one or more of the vascular risk factors was 61.6% at age 45-54, increasing to 68.2% by age 55-64 and 77.5% by age 65-74. The population attributable fraction of dementia from having one or more vascular risk factors increased from 22.7% (95% CI: 15.3-30.1%) for risk factors measured at age 45-54 to 26.4% (95% CI: 19.1-33.6%) at age 55-64 and 45.4% (95% CI: 32.5-58.3%) for risk factors measured at age 65-74. Our data indicate between 23%-45% of incident dementia cases in ARIC were attributed to a combination of hypertension, diabetes, or current smoking up through age 74. Public health strategies targeting modifiable vascular risk factors could delay or prevent new dementia cases.

